# Graphene-Based Nanomaterials as Heterogeneous Acid Catalysts: A Comprehensive Perspective

**DOI:** 10.3390/molecules190914582

**Published:** 2014-09-15

**Authors:** Bhaskar Garg, Tanuja Bisht, Yong-Chien Ling

**Affiliations:** 1 Department of Chemistry, National Tsing Hua University, Hsinchu 30013, Taiwan; E-Mail: bhaskargarg111@gmail.com; 2 Department of Chemistry, Government Degree College, Champawat 262523, Uttarakhand, India; E-Mail: tanuja.bisht16@gmail.com

**Keywords:** heterogeneous acid catalysis, nanomaterials, graphene, graphene oxide, sulfonation, sulfation, green chemistry

## Abstract

Acid catalysis is quite prevalent and probably one of the most routine operations in both industrial processes and research laboratories worldwide. Recently, “graphene”, a two dimensional single-layer carbon sheet with hexagonal packed lattice structure, imitative of nanomaterials, has shown great potential as alternative and eco-friendly solid carbocatalyst for a variety of acid-catalyzed reactions. Owing to their exceptional physical, chemical, and mechanical properties, graphene-based nanomaterials (G-NMs) offer highly stable Brønsted acidic sites, high mass transfer, relatively large surface areas, water tolerant character, and convenient recoverability as well as recyclability, whilst retaining high activity in acid-catalyzed chemical reactions. This comprehensive review focuses on the chemistry of G-NMs, including their synthesis, characterization, properties, functionalization, and up-to-date applications in heterogeneous acid catalysis. In line with this, in certain instances readers may find herein some criticisms that should be taken as constructive and would be of value in understanding the scope and limitations of current approaches utilizing graphene and its derivatives for the same.

## 1. Introduction

The prime source of energy on Earth for the present and foreseeable future is found in chemical bonds. Catalysis affords the means of changing the rates at which chemical bonds are formed and broken making it possible to elicit a desirable product over an undesired one, the phenomenon of chemical specificity. Among catalysis of substantial interest, acid catalysis is quite prevalent and probably the most routine operation in both industrial processes and academic research worldwide. The petroleum (biodiesel production as alternative fuels), chemical (production of most chemicals and materials), plastic (production of polymers and plasticizers), and pharmaceutical industries (production of new heterocycles as “wonder drugs”), essential to a healthy economy, rely heavily on acid catalysis. Not surprisingly, therefore, it lies at the heart of our quality of life.

In industry, with foremost focus on economic benefits, a large class of liquid-phase reactions are routinely executed with sulfuric acid (H_2_SO_4_), hydrofluoric acid (HF), and methanesulfonic acid (CH_3_SO_3_H) as bulk commodity catalysts. Unquestionably, the unique spatial separation (similar to that of enzymes) as well as the self-similarity of structures between the active sites in these homogeneous molecular acids not only allow consistent energetic interactions between each active site and reaction substrate (even at ambient conditions) but also make them responsive to most spectroscopic characterization techniques. Despite this, these catalysts require challenging processes for their separation from the homogeneous reaction mixture, resulting in abundant non-recyclable acid waste.

The shift towards an emphasis on catalyst recoverability and reusability has resulted in extensive research efforts devoted to the development of solid or heterogeneous acid catalysts, which are easier to separate, recover and reuse in desired reactions. In this milieu, endeavors have been made using, to name but a few, cation exchange resins (Amberlyst™-15, Nafion-H, *etc.*), solid superacids (SO_4_^2−^/ZrO_2_), (supported)metal oxides/sulfides/triflates (Al_2_O_3_, CdS, Yb(OTf)_3_), zeolites (H-ZSM-5), molecular sieves, silica, natural clays (bentonite, montmorillonite), and heteopolyacids to complement liquid-acid catalysts in a broad spectrum of acid-catalyzed reactions [1,2,3,4,5,6,7,8]. Although the mentioned solid catalysts certainly overcome some shortcomings, most of them are inappropriate owing to their deactivation (leaching or undesired deposits on active sites) [9,10], internal mass transfer (such as in zeolites) [11] or high mass transfer resistance (as in clay, molecular sieves *etc.*), unfavorable side reactions, high cost, and most importantly, toxicity issues associated with the presence of certain metals.

The pursuit of heterogeneous acid catalysts capable of meeting most of the abovementioned challenges has resulted in the consideration of carbon nanomaterials (CNMs), which can function as metal-free catalysts. Indeed, with the added advantage of classical nanofabrication techniques, the CNMs, which include mesoporous/amorphous carbon [12,13] and rich polytypes of carbon such as carbon nanotubes (CNTs), have produced considerable advancements in heterogeneous acid catalysis [14,15]. Notably, until the 20th century, the carbon family was only dominated by the well-known materials like graphite, diamond, fullerenes, and CNTs. However, this “aura” was lessened with the first isolation of free standing 2D graphene in 2004 [16] (in order to avoid unnecessary complexity to potential readers, we have adopted herein the common names currently in use in graphene-based catalysis research, yet we would also like to recommend the readers to consult the guest editorial published in the journal *Carbon* [17] for an exhaustive and productive account of the nomenclature of sp^2^ carbon nanoforms).

**Figure 1:**
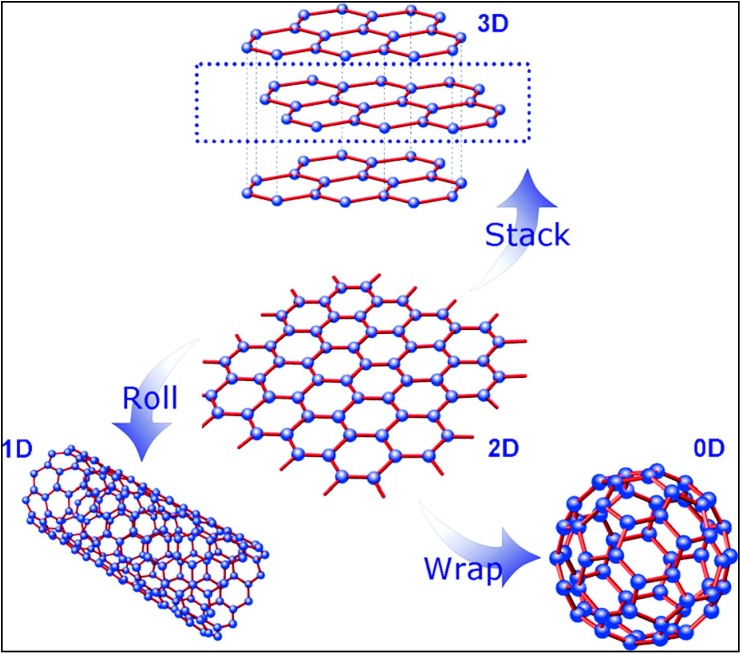
Graphene: the basic building block for other carbon allotropes, graphite (3D), fullerene (0D), and CNT (1D). Reprinted with permission from [18]. Copyright (2012) American Chemical Society.

Graphene could be considered as a potential building block of 3D graphite, 0D fullerenes (in a broader context or to some extent, it is reasonable to declare graphene as “mother” of all carbon nanostructures, as increasingly highlighted by many research groups; certainly, however, it is a misconception that graphene can produce fullerenes‒ notably, fullerenes have alternate pentagonal and hexagonal arrangements, whereas in graphene only hexagons should be present), and 1D CNTs (Figure 1) [18,19] and possess many exceptional properties that have surpassed those observed with its predecessor, CNTs. Not surprisingly, therefore, graphene-based nanomaterials (G-NMs) are (and will continue to be) increasingly used in optoelectronic [18], energy conversion and storage [18,20], photocatalysis/photovoltaic [21], switching and information storage [22], bacteria killing [23], biosensors [24,25] and other technological applications [26,27,28] in the imminent carbon age. On another front, the concept of carbocatalysis within the graphene framework is a relatively new but rapidly emerging research area at the interface of organic, material, green and sustainable chemistry [29].

It is worth commenting here that at certain instances, “carbocatalysis” can act as a bridge toward homogeneous catalysis (where both the reactants and catalysts are in a single phase) due to the existence of indefinitely persistent colloidal dispersions of carbocatalysts. In such cases, the process should be referred as “pseudo-homogeneous” because during the reaction, the catalyst is ostensibly in the same phase as reactants and products but can be recovered by means of centrifugation or membrane filtration after the reaction. Nevertheless, in order to avoid any confusion and also taking into account the recoverable features of G-NMs (lacking molecular entities), herein, we use the term “heterogeneous” throughout for graphene-based acid catalysis (G-AC).

After the seminal discovery by Bielawski and coworkers [30] of the use of graphene oxide (GO) as catalyst in alcohol oxidation, unquestionably, an appreciable range of G-NMs are currently available as catalysts for a variety of useful transformations. However, since the focus of this review article strongly lies on G-AC, interested readers are recommended to consult other review articles [29,31,32,33,34] for the corresponding unique and productive details.

The current state-of-the-art in G-AC can be largely categorized into graphite/graphene oxide (GO) and their sulfated (-O-SO_3_H) or sulfonated (-SO_3_H) derivatives. Whereas the former are obtained by chemical oxidation of natural graphite and/or subsequent exfoliation, the latter rely on the fundamental idea of surface functionalization of GO with sulfonating agents. Both classes exhibit unique acid activity and hold great promise in replacing traditional homogeneous and heterogeneous acid catalysts in an environmentally benign manner (Table 1). Thus far, to the best of our knowledge, G-AC has not been reviewed by any research group and we would like to be the first to shed light on this fast-growing field.

**Table 1:** A comparison between the characteristic features of sulfuric acid, Amberlyst™-15 and G-NMs in acid catalysis.

Features	Sulfuric Acid	Amberlyst™-15	G-NMs
Physical state	*Liquid*	*Solid*	*Solid*
Acid activity	*High*	*High*	*High*
Surface Area	*-*	*Relatively low*	*High*
Operating conditions	*Typically harsh*	*Relatively Mild*	*Variable* *^a^*
Diffusion/Leaching	*None*	*Possible*	*Negligible*
Recoverability	*Difficult*	*Easy* *^b^*	*Easy* *^b^*
Reusability/cycles	*Difficult*	*Easy/Less cycles* *^c^*	*Easy/More cycles* *^c^*
Water tolerance/stability	*High*	*Relatively low*	*High*
Cost-effectiveness	*Cost-effective*	*Relatively expensive*	*Variable* *^a^*
Mass transfer	*-*	*Relatively low*	*High*
Reaction selectivity	*High*	*Variable* *^a^*	*Variable* *^a^*
Eco-friendly use	*No*	*Yes*	*Yes*
Industrial use	*Frequent*	*Selected* *^d^*	*Awaiting*

*^a^* Depending upon the types of reactants, catalyst, and nature of reactions; *^b^* Amberlyst™-15 can be recovered simply by filtration through a common filter paper while G-NMs are efficiently recovered either by membrane filtration or centrifugation; *^c^* The number of cycles, herein, should be taken as (partial)loss of acidic sites; the less cycles symbolize significant loss in acid activity while due to no or partial loss in acid activity, G-NMs can be used repeatedly; *^d^* Amberlyst™-15 is commercially used at industrial scale in the addition of alcohols to alkenes to form ethers for gasoline boosters.

As such, this review will begin with a brief discussion on the synthesis and structure of GO and its precursor, graphite oxide. Then, we will focus on unconventional structural models that have emerged only recently and are ascribed to be responsible for the inherent acidic properties of graphite oxide/GO. Afterwards, we will focus on the description of various synthetic routes to obtain sulfonated and sulfated analogues of graphene. In this regard, systematization according to the type of reducing and sulfonating agents is established. An equal emphasis is placed while discussing the properties and characterization data of G-NMs based on elemental analysis (EA) and Raman spectroscopy. Lastly, the catalytic applications of G-NMs are categorized based on: (i) graphite oxide; (ii) GO; and (iii) sulfated or sulfonated derivatives and discussed in conjunction with other acid catalysts. It should be noted that in some applications, both GO and its sulfonated counterparts have been examined together and thus the organization of this review should be regarded as a flexible guide to the classification of these applications.

## 2. Graphite Oxide and Graphene Oxide (GO)

### 2.1. Synthesis and Structure

Historically, graphite oxide was discovered as a way to measure the molecular weight of graphite. Indeed, this was the intention of Brodie when in 1859 [35] he heated a mixture of graphite and fuming nitric acid in the presence of potassium chlorate. The repeated oxidation (four replicates) of the as-obtained product afforded the very first sample of graphite oxide as a light yellow solid which was termed “graphic acid”, based on its dispersion behavior at different pH values. In a subsequent follow-up work in 1898 [36,37], Staudenmaier improved the oxidation protocol by adding potassium chlorate in small portions and acidifying the mixture with concentrated H_2_SO_4_, which completely avoided the four repetitions of the oxidation steps and was thus ascertained more practical. Over the span of 60 years, in 1958 [38], Hummers introduced potassium permanganate as an oxidant in a mixture of sodium nitrate and concentrated H_2_SO_4_ resulting in a more heavily oxygenated form of graphite oxide. This protocol was proved safer as it not only allowed *in situ* generation of nitric acid but also avoided the use of highly corrosive fuming nitric acid. In a bid to replace *in situ* production of nitric acid with less corrosive phosphoric acid, a new oxidation protocol (Tour’s method) is reported recently [39], yet Hummers method has been well appreciated among scientific community and increasingly adopted to date by many researchers worldwide.

Aside from the operative oxidation procedures, the chemical structure of graphite oxide has been the subject of extensive research over the years. Consequently, several structural models of graphite oxide have been surfaced from time to time. In this context, Hofmann-Holst [40], Ruess [41], Clauss [42], Scholz-Boehm [43], Nakajima [44], Nakajima-Matsuo [45], Lerf-Klinowski (LK) [46,47,48], Dékány [49], and Ajayan [50] models are especially notable. However, due to the variable oxidation procedures leading to sample-to sample variability, nonstoichiometric atomic composition and lack of a “hallmarked” characterization technique, the precise chemical structure of graphite oxide remains elusive and still a subject of further discussion. Overall, the LK model [48] of graphite oxide is perhaps the most popular and widely accepted among graphene research community.

As such, it is assumed that the basal plane of heavily oxygenated graphite oxide is highly populated with hydroxyl (-OH) and epoxide (C-O-C) functions, while the edge-plane consists of carbonyl (C=O) and carboxylic acid (-COOH) groups. The coverage of the oxygen-rich functional groups in graphite oxide substantiates hole defects (nanovoids and vacancies) throughout the surface and deviates it from the state of pristine graphite. Indeed, the presence of such features on the graphite oxide surface has recently been observed [51] using high-resolution transmission electron microscopy (HRTEM).

**Figure 2:**
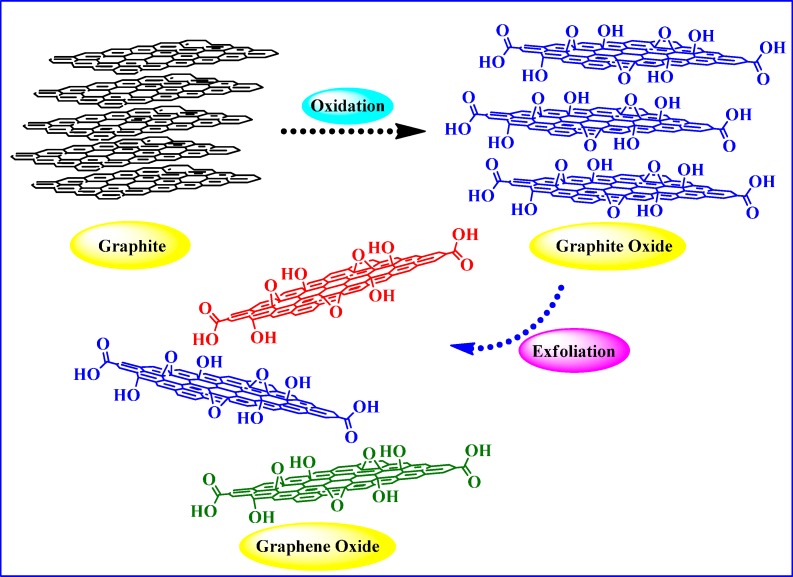
Synthesis of graphite oxide and graphene oxide (GO) from natural graphite.

Graphite oxide is chiefly composed of carbon, oxygen, and hydrogen atoms retaining a C/O ratio in between 1.8 to 2.5, while the most common C/O ratio for different samples is ~2 [40,42,49,52]. In this context, it should be noted that the stoichiometric ratio between the constituent elements of graphite oxide is not fixed and varies depending upon the oxidation level. Consequently, regardless of the synthesis protocol followed [36,38] the composition and structure of graphite oxide do not change significantly even after the addition of excess oxidizing agent. GO, an exfoliated form of graphite oxide, is another important intermediate between graphite and graphene and usually attained via mechanical (vigorous stirring or ultrasonication), chemical (ionic liquids) or thermal treatment of graphite oxide. At this juncture, it is critical to understand the technical point of differentiation between graphite oxide and GO. Whereas graphite oxide represents the lamellar structure (consisting of layers of carbon from the original graphene lattice), GO largely exists as mono-, bi- or at most a few layers (3 to 4) of graphene sheets. Nevertheless, despite being structurally different, both are chemically similar, having a complex cocktail of oxygen functionalities (Figure 2) rendering them hydrophilic in nature. As a consequence, water molecules can readily intercalate between the layers resulting in an increase of the interlayer distance and change of hybridization state (in oxidized carbons) from sp^2^ (planar) to sp^3^ (tetrahedral).

### 2.2. Acidic Properties of Graphite Oxide and GO

Aqueous solutions of graphite oxide have significant acidity, with a pH in between 3 to 4. It has long been demonstrated that 100 g of graphite oxide contains 500–800 mmol of active acid sites [40,42] *i.e.*, one acid site is present for every 6–8 carbon atoms that can participate in cation exchange reactions. In order to explain such high acidity of graphite oxide, Clauss *et al.* [42] suggested the presence of enolic groups and proposed the empirical formula C_8_O_2_(OH)_2_ for the composition of graphite oxide. However, the authors were unable to provide any experimental data in support of this suggestion.

In yet a more recent scenario, it is commonly assumed that graphite oxide obtained by the (modified)Hummer’s method [38,48] contains lots of recoverable Brønsted acid sites (though benchmark experimental evidences are still in question) including -OH and -COOH groups on the basal plane and flake edges, respectively. Accordingly, the acidic character of graphite oxide is likely due to the synergistic effect of these functionalities. However, controlled experiments by Dhakshinamoorthy *et al.* [53] with GO, H_2_SO_4_, *p*-toluenesulfonic acid (*p*-TSA), and glacial acetic acid have clearly indicated that the active acid sites of GO are not indeed -COOH groups but probably HSO_4_ groups as evidenced by elemental analysis (1.16 wt % of sulfur atoms). In fact, the thermal treatment of GO at 200 °C removed most of the HSO_4_ groups and resulted in catalytically inactive, partially reconstituted, GO having a negligible sulfur content. A similar observation (at relatively high temperature; 400 °C) has also been reported recently while using GO as an acid catalyst in the conversion of carbohydrates into 5-ethoxymethylfurfural [54].

In an elegant work, Dimiev *et al.* [55] have recently proposed a new explanation for the graphite oxide acidity that may allow one to add critical details to existing structural models of graphite oxide with higher confidence. It is well known, as increasingly highlighted in most published work, that graphite oxide has a light yellow appearance during its first quenching with water. However, with cumulative water washings, this color gradually changes from light yellow to dark brown or even black, which indicates that significant chemical transformations occur in the course of the purification of graphite oxide. Taking this into account, the group [55] decided to avoid exposing the graphite oxide material to water. Thus, by introducing non aqueous solvents such as methanol, isopropanol, ethyl acetate and glacial/trifluoro- acetic acid during the quenching and purification steps of graphite oxide, they synthesized different samples of pristine graphite oxide (pGO) having a significant amount of vacancy defects terminated by ketone groups. The products were extensively characterized by Fourier transform infrared spectroscopy (FTIR), UV-visible and X-ray photoelectron spectroscopy (XPS), thermogravimetric analysis (TGA), ^13^C solid state nuclear magnetic resonance (SSNMR), and scanning electron microscopy (SEM).

**Scheme 1:**
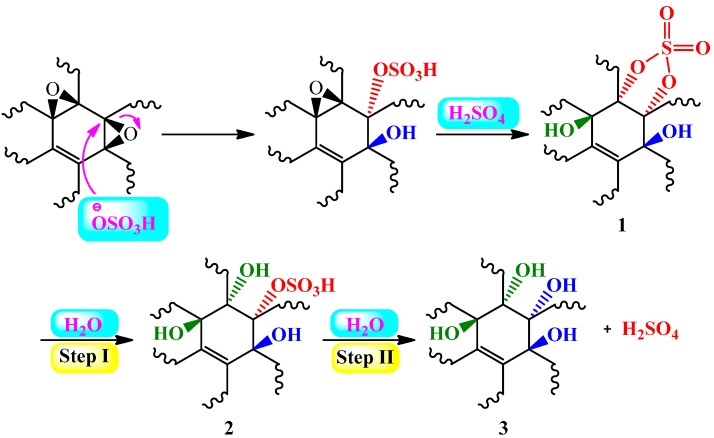
Hydrolysis of cyclic sulfate **1** into hydrosulfate **2** (Step I) and sulfuric acid (Step II). Adapted with permission from [55]. Copyright (2012) American Chemical Society.

It was postulated that the oxidized areas (sp^3^) of as-synthesized pGO are dominated by epoxide groups while covalent sulfates (primarily existing as cyclic sulfates **1**) and -OH groups are present in small amounts. When pGO was exposed to water, hydrolysis occurred in two steps. In the first step, cyclic sulfates are cleaved to monosulfate **2**, while 1,2-diol **3** and H_2_SO_4_ are formed in the second step of hydrolysis (Scheme 1). The as-produced **2** and H_2_SO_4_ were ascribed to be responsible, in part, for the highly acidic properties of graphite oxide. Furthermore, as evidenced by experimental data, it was proposed that acidic properties of graphite oxide are significantly affected by stepwise conversion of tertiary alcohols into edge-pane ketones, which remain in equilibrium with their hydrated form.

**Scheme 2:**
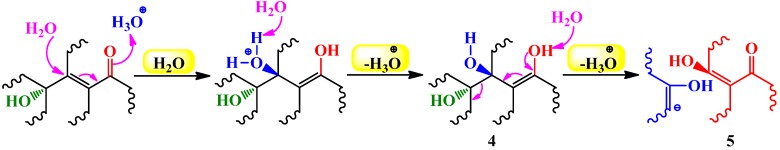
A schematic representing the formation of vinylogous carboxylic acid **5** via C-C bond cleavage of enol **4**, which in turn was obtained via nucleophilic attack of water molecule on a GO fragment having one hydroxyl and ketone group. Adapted with permission from [56]. Copyright (2013) American Chemical Society.

The group further examined the effect of water on GO acidity and proposed an unconventional view of GO chemistry [56], a “dynamic structural model” (DSM). As per this theory, “GO does not exist as a static structure with a given set of functional groups, but rather generates them and constantly changes its structure through interaction with water”. As such, the GO acidity was brought about by C‑C bond cleavage, formation of vinylogous carboxylic acid **5** (rendering the -OH groups acidic) (Scheme 2), and the generation of protons. In this milieu, it should be noted that the formation of an electrical double layer at the GO interface in aqueous solutions is the main driving force for the observed GO acidity. Equally important is to understand that on prolonged exposure to water, GO flakes gradually degrade and attain a humic acid-like structure.

**Figure 3:**
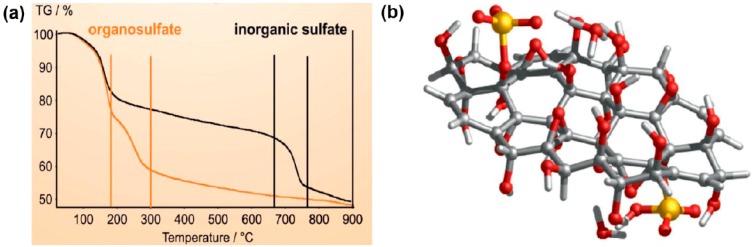
(**a**) The TGA data of GO-NaHSO_4_ revealing two main decomposition steps for sulfur-containing species. (**b**) The proposed structural model of GO terminated with epoxy, hydroxyl, and organosulfate as dominant functional groups. Reproduced with permission from [57]. Copyright (2013) Wiley-VCH Verlag GmbH & Co.

In a more recent report, Eigler *et al*. [57] undertook a systematic study in order to determine the sulfur species in GO and its base-treated counterparts, the so called GO-NaOH, GO-NaHSO_4_, and GO-Na_2_SO_3_. In contrast to the previous work on graphite oxide hydrolysis [55], the group postulated that only the first step of hydrolysis takes place, while mono/organosulfates **2** are fairly stable and remain bound on GO (see Scheme 1), a key aspect for graphite oxide/GO acidity. As evidenced by thermogravimetry-mass spectroscopy (TGA-MS), it was concluded that organosulfates are the integral part of any GO and decompose at about 250 °C. On the other hand, the weight loss step in between 700–800 °C could be attributed to the pyrolysis of inorganic sulfur as sulfates or sulfite in GO-NaHSO_4_, and GO-Na_2_SO_3_, respectively (Figure 3a). Moreover, a TGA-MS analysis using sodium dodecylbenzenesulfonate (decomposition temperature of ~500 °C) as a reference completely excluded the presence of sulfonic acid groups on the GO surface. Accordingly, the structural model of GO was extended by adding organosulfate in addition to epoxy, and hydroxyl groups, which are predominantly located above and below the carbon skeleton (Figure 3b). It was estimated that, in addition to hydroxyl and epoxy groups, one organosulfate group per 20 carbon atoms is present in GO and must be located on both sides of carbon lattice as well as the edges of GO flakes. The observation brought about by this study that the basic washing procedures could apparently be effective in GO purification, especially in removing sulfur species, is quite appealing.

In light of the above discussion, it may be concluded that -COOH groups have nothing to do with the acidic nature of graphite oxide/GO while the presence of sulfur as organosulfates, an integral part of oxidation protocols, is largely responsible for the observed acidity. As a final note, we anticipate that the exhaustive characterization of GO is a prerequisite prior to surface functionalization, especially, in sulfonation reactions as the presence of organosulfates in GO may significantly influence the characterization data of the resulting NMs and lead to misinterpretations.

## 3. G-NMs *vs.* Sulfonation and Sulfation

**Scheme 3:**
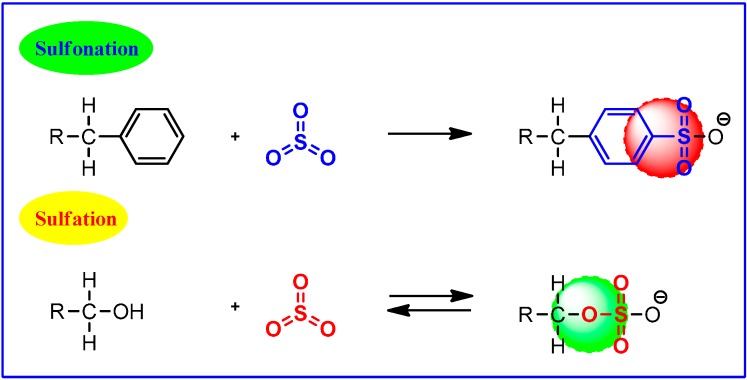
Sulfonation *vs.* sulfation.

The integration of sulfonic acid functional groups (-SO_3_H) into CNMs is well known and emerged as a powerful strategy to produce promising solid acids for a range of acid-catalyzed reactions [12,13,14,15,58,59]. However, the sulfonation chemistry of G-NMs has developed only recently and continues to expand for diverse applications. At this juncture, it is critical to differentiate between sulfonation and sulfation reactions (Scheme 3) as the two are often confused but the structural differences are quite significant, making the use of separate terms more appropriate.

In sulfonation, sulfur trioxide (SO_3_), the active species, reacts for example with an alkyl benzene and forms a C-S bond. Notably, the resultant alkyl benzenesulfonic acid is a stable molecule. Sulfation, on the other hand, involves the formation of a C-O-S bond and the resultant alcohol sulfuric acid is somewhat less stable. Unless neutralized, it decomposes to form sulfuric acid and the starting alcohol. This stability difference in the products of SO_3_ reaction also has an intense impact on the choice of process used to produce sulfonates or sulfates.

In general, graphene-based sulfonation can be achieved in two steps: (a) reduction of GO to reduced graphene oxide (rGO) and (b) sulfonation of rGO. However, the direct sulfonation of GO and two-steps reduction protocols have also been undertaken at certain instances. The concrete methods, reducing and sulfonating agents are classified, summarized, and discussed below.

### 3.1. Reduction of GO

To date, one of the most important reactions of GO is its reduction leading to rGO, similar to that of pristine graphene. Although the reduction of GO can be achieved through thermal/H_2_ [60,61,62], electrochemical [63], or chemical means [19,64,65,66,67,68], the latter is widely used in the graphene research and will only be discussed here. The emphasis is given to C/O ratio as well as Raman spectroscopy.

The aqueous colloidal dispersions of GO can be effectively reduced by a range of reducing agents having an established background in synthetic chemistry (see the recent review [69] for a comprehensive table of reducing agents for GO). The conversion of GO into rGO is usually achieved by an increased aggregation or hydrophobicity (due to the removal of oxygen containing groups) manifested in a color change from brown to black. Based on elemental analysis (EA), a decrease in elemental oxygen contents thereby resulting an increase of C/O ratio certainly signify the efficacy of the employed reducing agents.

Raman spectroscopy is a handy tool to characterize the structure and quality of G-NMs including graphite, graphite oxide, GO, rGO, and other carbonaceous materials [70,71]. In this context, the two most prominent features in (r)GO are the D and G vibrational bands (1100–1800 cm^−1^). Whereas the former gives an idea about ordered or disordered structure of carbon, the latter is an indicator of the stacked carbon domain. Also of interest is the 2D band, a dominant sharp peak always observed for single layer graphene. It has been demonstrated that Raman scattering can be used as a fingerprint for single, bi, and a few layer graphene [72,73]. The ratio of intensities of two bands (*I*_D_/*I*_G_) can be used as a means of determining the structural changes within graphene samples. For instance, a high *I*_D_/*I*_G_ ratio clearly indicates a certain amount of disorders/edges or exfoliation [71].

In general, graphite displays a prominent G peak at 1580 cm^−1^, attributable to the first-order scattering of the E_2g_ mode. On the contrary, GO shows two peaks at 1363 and 1594 cm^−1^ corresponding to the D and G bands, respectively. The G band is associated with the vibrations of sp^2^ carbon atoms in the 2D graphitic lattice whereas the D band is related to the vibrations of sp^3^ carbon atoms of defects or disorder as mentioned above [71,73]. After the reduction, the *I*_D_/*I*_G_ ratio changes considerably, as will be discussed below taking hydrazine (N_2_H_4_), sodium borohydride (NaBH_4_), and lithium aluminum hydride (LiAlH_4_) as representative reducing agents.

#### 3.1.1. Hydrazine (N_2_H_4_)

N_2_H_4_ is known to scavenge oxygen while itself being broken down into water and nitrogen, making it an attractive option where most reducing agents exhibit moderate to strong reactivity toward water. N_2_H_4_·H_2_O was firstly used as reducing agent by Stankovich *et al.* [74] in an effort to obtain individual sheets of graphene from GO. Under the optimized conditions, the number of polar functionalities on GO’s surface was decreased significantly and resulted in aggregated layers of graphene sheets, rGO. Based on the extensive characterization data, it was proposed that the reduction of GO proceeds via a nucleophilic attack of N_2_H_4_ on an epoxide function. The EA results were in accord to expectations and a high C/O ratio (10.3/1) was measured for rGO over GO (2.7/1). Likewise, the Raman spectra indicated an increase in *I*_D_/*I*_G_ ratio of as-obtained rGO which was suggested to be the result of a decrease in the size of sp^2^ carbon domains (by the formation of sp^3^ carbons) upon reduction. Despite its good efficiency, the severe toxicity coupled with the introduction of heteroatomic impurities somewhat limits the practical use of N_2_H_4_.

#### 3.1.2. Sodium Borohydride (NaBH_4_)

NaBH_4_ is one of the most used hydrides in research laboratories, as it can be readily solubilized in aqueous and alcoholic media. The first example of NaBH_4_ usage in GO reduction was reported by Muszynski *et al.* [75] to achieve the physisorption of Au nanoparticles on amine grafted graphene. Later on, Gao *et al.* [50] performed the total reduction of GO using NaBH_4_ as the first of a three-step protocol. The C/O ratio of as-obtained rGO was almost twice (4.78) that of GO (2.44). In addition, the Raman spectra of rGO exhibited an increased *I*_D_/*I*_G_ ratio (1.91) compared to GO (0.95), which is consistent with what is observed with N_2_H_4_, as mentioned above. Unlike N_2_H_4_, NaBH_4_ generates additional alcohols as principal impurities [76], a possible cause of a low C/O ratio relative to that of N_2_H_4_.

#### 3.1.3. Lithium Aluminum Hydride (LiAlH_4_)

LiAlH_4_ (LAH) is another strong reducing agent of the hydride family that remained unused in GO reduction for a long time, presumably, due to its strong reactivity towards water (production of H_2_ gas) and capability to reduce carbonyl, epoxy, ester, and carboxylic functions (unselective nature) to hydroxyl groups. The first LAH-triggered reduction of GO, in conjunction with N_2_H_4_ and NaBH_4_, was reported by Ambrosi *et al.* [77]. The strong reducing strength of LAH was evidenced by a highest C/O ratio (12) while the reduction of GO (C/O ratio 3.4) with N_2_H_4_ and NaBH_4_ provided relatively low C/O ratios of 11.5 and 9.5, respectively. The FTIR spectrum of as-obtained rGO indicated the presence of -CH_2_ groups due to the alkene hydrogenation of unsaturated carbonyls. The GO obtained by graphite oxidation provided a considerably high *I*_D_/*I*_G_ ratio (1.10) and was postulated to be due to the extensive disorder in the graphitc structure. On the contrary, upon reduction, the *I*_D_/*I*_G_ ratio decreased to 0.61, 0.97, and 0.63 for NaBH_4_, N_2_H_4_ and LAH, respectively. A decrease in *I*_D_/*I*_G_ ratio clearly indicates that the disorder associated with the amorphous GO diminishes. There are a handful of other characterization techniques (XRD, XPS, EDS elemental mapping) for differentiating GO and rGO. Besides this, the AFM can be used to determine the single layer morphology of the desired sample. We recommend that readers consult the original literature for additional details, as mentioned previously.

### 3.2. Sulfonation of rGO and GO

As discussed above, the chemical reduction of oxygen functionalities on GO sheets produces rGO with the reintroduction of large aromatic domains. Consequently, rGO has increasingly used for the non-covalent adsorption of polymers [78,79] and aromatic species [80,81] via π–π stacking and van der Waals interactions. On the other hand, the covalent functionalization of rGO is quite uncommon and not performed significantly. In this milieu, the surface modifications of (r)GO with -SO_3_H groups have provided a handle to narrow down this huge gap. This section will deal the concrete methodologies used for sulfonation reactions of (r)GO along with a brief on characterization data. For the sake of clarity, sulfonation reactions are classified according to the type of sulfonating agents (Table 2).

**Table 2:** List of sulfonating agents for (r)GO to G-AC.

Sulfonating Agents	Reducing Agents ^a^	Reaction Conditions ^b^	Acid Density ^c^	Applications ^d^	Ref.
*4-Benzenediazonium sulfonate*	N_2_H_4_·H_2_O	EtOH/H_2_O, H_3_PO_2_, 3–5 °C, 1.5 h	1.55 mmol H^+^ g^−1^	Hydrolysis of ethyl acetate	[82]
*Fuming sulfuric acid*	NaBH_4_	Autoclave, 180 °C, 24 h	1.2 mmol H^+^ g^−1^	(1) Esterification reactions (2) Peckmann reaction (3) Hydration reaction	[83]
*Chlorosulfonic acid*	*n*-butyl lithium	ClSO_3_H, 0 °C, RT ^e^, overnight, NaOH, HCl, H_2_O	-	Ester exchange reactions	[84]
*4-Benzenediazonium sulfonate*	NaBH_4_	0 °C, 2 h, Hydrazine, 100 °C, 24 h	-	Synthesis of xanthenes and benzoxanthenes	[85]
*Chlorosulfonic acid*	-	CHCl_3_, 70 °C, 4 h	1.2 mmol H^+^ g^−1^	Chemical conversion of biomass derived carbohydrates	[86]
*4-Benzenediazonium sulfonate*	(i) NaBH_4_ (ii) N_2_H_4_	RT ^e^, overnight	0.5–1.7 mmol H^+^ g^−1^	Dehydration of xylose to furfural	[87]
*Ammonium sulfate*	-	235 °C, 30 min, argon atmosphere	EW ^f^ = 725 ± 5 g/mol	As support for metal nanocatalysts	[88]
*Sulfuric acid*	Microwave irradiation, 190 °C	160 °C, 5 h nitrogen atmosphere	1.9 mmol H^+^ g^−1^	Production of biofuels	[89]

^a^ List of reducing agents used in sulfonation methods of GO and partially reduced GO; ^b^ Sulfonation carried out in aqueous medium or pure solution of the sulfonating agents unless stated otherwise; ^c^ Determined by acid-base titrations; ^d^ Applications in metal-free heterogeneous acid-catalyzed reactions except ref. ([88,90], the latter is not included in the table); ^e^ Room temperature; ^f^ Equivalent weight.

#### 3.2.1. Diazonium Salt of Sulfanilic Acid; 4-Benzenediazonium Sulfonate

Diazonium salts represent a class of organic compounds having a general formula of RN_2_^+^ X^−^ where R may be an alkyl or aryl residue and X is an (in)organic anion. The diazonium salts having an aryl group are usually stable in aqueous solutions at temperatures below 5 °C (the N_2_ group does not tend to be lost as nitrogen gas) and serve as important intermediates in the organic synthesis of azo dyes [91,92,93,94]. The nitrosation of primary aromatic amines with HNO_2_, which itself generated *in situ* from NaNO_2_ and a mineral acid, is the most common method for the preparation of diazonium salts. However, being non-nucleophilic, the diazonium tetrafluoroborate salts can be isolated and are stable at room temperature. The process of forming diazonium compounds has been given a variety of names, including diazotation, diazoniation, and diazotization.

**Scheme 4:**
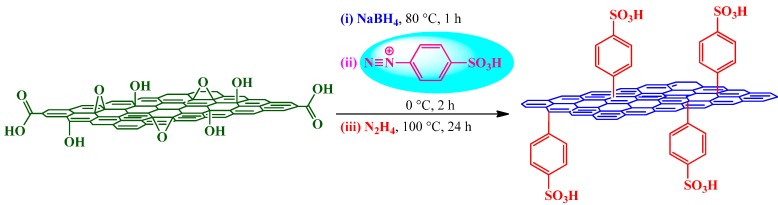
Sulfonation of GO with 4-benzenediazonium sulfonate.

The usage of diazonium chemistry in the graphene domain was first reported by Lomeda *et al.* [95] to achieve the high dispersibility of graphene platelets in a range of polar aprotic solvents. However, the first attempt towards graphene sulfonation was performed by Si and Samulski [96] following a three step protocol as shown in Scheme 4. It was postulated that prereduction (step 1) is crucial both to enable sulfonation reaction (step 2) and to achieve complete reduction (step 3). The EA indicated that after prereduction of GO, a S/C ratio of 1:35 could be achieved via diazotization. However, only a 1:148 ratio is possible without step 1. The as-obtained sulfonated graphene exhibited an electrical conductivity of 1250 S m^−1^ implying that an extended conjugated sp^2^ network is restored in the sulfonated graphene.

On another note, Ji *et al*. [82] introduced a two-step protocol avoiding the prereduction step. Specifically, GO was first reduced with N_2_H_4_·H_2_O and dialyzed for 2 days. The diazotization of sulfanilic acid followed by sulfonation of purified rGO with 4-benzenediazonium sulfonate in the presence of hypophosphorous acid (H_3_PO_2_) afforded sulfonated graphene (S-graphene) having crumpling features. The solid state ^13^C-NMR spectrum of S-graphene exhibited a small peak at 136 ppm, which was assigned to the carbons in the covalently attached phenyl-SO_3_H groups. The Raman spectra of S-graphene indicated a slightly higher *I*_D_/*I*_G_ ratio than that of rGO, which was likely a result of introduction of abundant -SO_3_H groups to the sp^2^ carbon network. It was further evidenced by EDS mapping where sulfur was found to be homogeneously distributed on the whole surface. The neutralization titrations of S-graphene indicated an ion exchange capacity (IEC) of 1.55 mmol H^+^ g^−1^, which was significantly higher than commercially available Nafion NR50 (0.80 mmol H^+^ g^−1^) solid acid catalyst.

Following Samulski’s approach, Zhao *et al.* [97] obtained highly dispersed sheets of sulfonated graphene (SG) and applied them as adsorbents for persistent organic aromatic pollutants such as naphthalene and 1-naphthol from aqueous solutions.

A similar, three-step preparation method was also carried out by Lam *et al.* [87] during their investigations on xylose dehydration. The BET surface area of resulting NMs was measured to be 318 (GO), 680 (SGO), and 634 (SG) m^2^·g^−1^; quite higher than that of Hu’s work, even though the S/C ratio was nearly comparable (1:135 *vs.* 1:140) for SG. Of further interest is the clear discrepancy in the Raman data. The *I*_D_/*I*_G_ ratio was increased from GO (0.89) to SG (1.00) in the previous work [97], whereas a significant decrease was observed in *I*_D_/*I*_G_ ratio from GO (1.16) to SG (0.78) in the latter [87]. However, the product of step 2 (SGO; obtained after prereduction of GO followed by sulfonation) showed a slight increase in *I*_D_/*I*_G_ ratio from GO (1.16) to SGO (1.27). Although no explanation has been given for this difference, we assume that the synthesis and purification steps of graphite oxide or GO, as mentioned previously, can significantly affect its further functionalization and experimental properties. Indeed, this assumption may be linked to the EA analysis in the same work. Surprisingly, all three GO, SGO, and SG NMs indicated the presence of 0.6% of sulfur, even though their IEC values were significantly different; 2.0, 1.7, and 0.5 mmol H^+^ g^−1^ for GO, SGO, and SG, respectively. This data suggests that the presence of oxygen-functionalities, in particular, -OH groups are responsible for the acidity of G-NMs, which indeed is a misconception as mentioned above.

Zhang *et al.* [98] prepared SG sheets as described above [96] and used them as a sorbent in micro-solid-phase extraction (µ-SPE) coupled with gas chromatography-mass spectrometry for the determination of polycyclic aromatic hydrocarbons in water. Based on Samulski’s pioneering work [96] on GO sulfonation, a number of other groups have also prepared SGs and used them for different applications [85,99,100].

#### 3.2.2. Fuming Sulfuric Acid

Fuming sulfuric acid or oleum commonly refers to a solution of sulfur trioxide (SO_3_) dissolved in sulfuric acid. It can be described by the general formula H_2_SO_4_·*x*SO_3_ where *x* is the free SO_3_ molar content and is often known as 30% oleum. It is produced in the contact process in which S is oxidized to SO_3_ and subsequently dissolved in concentrated H_2_SO_4_. Owing to its high enthalpy of hydration, it serves as a central intermediate in the production of H_2_SO_4_. In typical organic chemistry, oleum is used in the nitration of nitrobenzene and the manufacture of explosives. Due to its relatively low cost (~US$ 0.153 per pound of reactive SO_3_), oleum sulfonation is one of the industrially preferred strategies, especially, for versatility in feedstock selection to build materials that are truly application specific. In general, oleum sulfonation is an equilibrium and hydrothermal process, which can be operated both as a batch and continuous process.

**Scheme 5:**
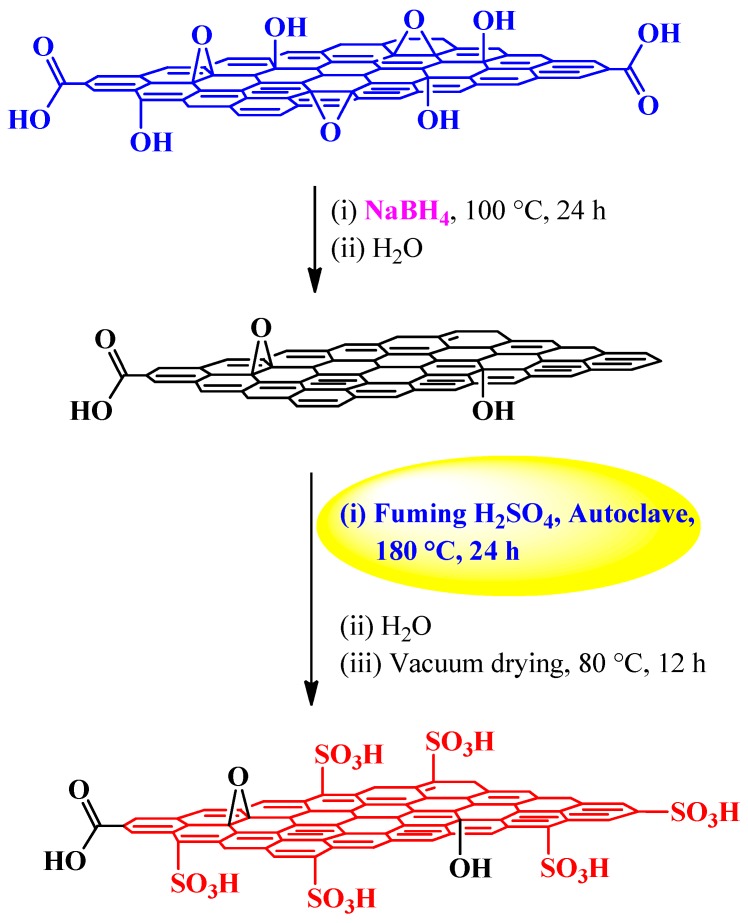
Oleum-assisted sulfonation of GO into G-SO_3_H.

In graphene research, the oleum-assisted sulfonation was first achieved by Liu *et al.* [83] by following the route shown in Scheme 5. The resulting material was termed sulfated graphene (G-SO_3_H). However, we would like to rename it sulfonated graphene, which is more appropriate as there is C-S bonding rather than O-S. The TEM and AFM images indicated the sheet like structure of G-SO_3_H with an average thickness of 0.8–3.0 nm. Compared with GO (0.89), G-SO_3_H gave a relatively high *I*_D_/*I*_G_ ratio (1.02), which can be attributed to a decrease in the average size of the sp^2^ domain as described above. The high resolution S2p XPS spectrum of G-SO_3_H displayed a peak at 168.3 eV (associated with S-O bonds); rather lower than those of SO_3_H-functionalized ordered mesoporous carbon (OMC-SO_3_H; 168.8 eV) and Amberlyst-15 (168.9 eV), which was likely a result of electron transfer from graphene to the -SO_3_H groups [16]. In this milieu, it is important to stress that S2p binding energy is a direct measure and quite sensitive to the acidic strength [101].

The thermal stability of G-SO_3_H was evaluated by TGA in association with OMC-SO_3_H. Specifically, G-SO_3_H revealed two main decomposition steps, centered at 268 and 568 °C, which were assigned to the degradation of -SO_3_H groups and carbon framework, respectively. In contrast, weight loss temperatures in OMC-SO_3_H were centered at 237 °C and 390 °C for -SO_3_H groups and carbon framework, respectively. The better stability of carbon framework and -SO_3_H groups in G-SO_3_H over OMC-SO_3_H was attributed to its non-amorphous characteristic leading to stronger interaction with -SO_3_H groups.

It would be worthwhile to remind readers again here that TGA results are in sharp contrast to Hirsch’s experimental findings [57], where it is shown that -SO_3_H groups dissociate at ~500 °C. Furthermore, the observed weight loss at 268 °C in G-SO_3_H is quite close enough to that of organosulfates, which may be attributed to the presence of sulfur species in the parent GO. The acid content of G-SO_3_H was evaluated by acid-base titration and was found closer (1.2 mmol H^+^ g^−1^) to OMC-SO_3_H (1.3 mmol H^+^ g^−1^) but far below that of Amberlyst-15 (4.7 mmol H^+^ g^−1^).

While diazotization-based GO sulfonation involves tedious multi-step synthesis, oleum-assisted hydrothermal sulfonation does not, making it an attractive strategy for sulfonation of GO. Despite this, the use of autoclaves with the added disadvantage of relatively high temperature conditions (≥180 °C) make this process quite expensive as well as energy inefficient. With these realizations, we decided to reinvestigate this reaction under relatively mild conditions. Indeed, combined with a knowledge of acid functional ionic liquids [102,103,104] and their exceptional product-switching ability in tetrapyrrolic organic systems [102], we were able to synthesize sulfonated graphene with simple processing and handling, one of the important tenets of “green chemistry”. In particular, the oleum-assisted hydrothermal sulfonation of rGO in a standard fume cupboard at 120 °C for 3 days afforded sulfonated graphene exhibiting an IEC of 1.55 mmol H^+^ g^−1^. The as-obtained sulfonated graphene was successfully used in the synthesis of ester plasticizers, and other industrially important chemicals. The details of these findings will be reported elsewhere.

**Scheme 6:**
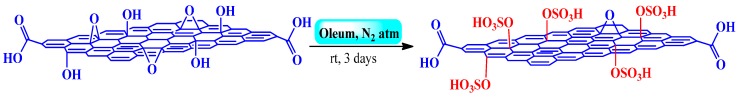
Oleum-assisted sulfation of GO into GO-OSO_3_H.

In yet another work, Liu *et al.* [90] used oleum to develop single-layer sheets of sulfated GO (GO-OSO_3_H) (though oleum-assisted sulfation is not commonly practiced) as a hole-extraction layer in high performance polymer solar cells. It was postulated that on treatment with oleum, epoxy and hydroxyl groups on the basal plane of GO are either removed or transformed into sulfate groups as shown in Scheme 6. This is quite reasonable because strong acids [50,105] such as oleum exhibit a high dehydration tendency and can lead to the partial reduction of GO. The oleum-induced basal plane reduction of GO was evidenced by comparing the XRD patterns for GO and GO-OSO_3_H. In particular, a peak shift from 2θ = 11.50° (*d* = 7.65 Å) for GO to 2θ = 26.40° (*d* = 3.91 Å) was recognized for GO-OSO_3_H, a characteristic feature of partially restacked graphene domain. These results were further verified by XPS, UV-visible absorption, and Raman spectroscopy, the latter of which showed a slightly higher *I*_D_/*I*_G_ ratio for GO-OSO_3_H (0.88) than that for GO (0.78).

The acid density of GO-OSO_3_H was evaluated to be 5.86 mmol H^+^ g^−1^, which was relatively higher than that of GO (3.15 mmol H^+^ g^−1^). Such a high acidity (even higher than that of Amberlyst™-15) of GO-OSO_3_H was attributed to both the presence of -OSO_3_H and -COOH groups and may be linked to high S content (4.49 wt %) as determined by EA. Furthermore, the acidity of GO was interpreted in terms of -COOH and -OH groups, however, no EA data was presented by the authors [90].

#### 3.2.3. Chlorosulfonic Acid

Chlorosulfonic acid (ClSO_3_H) or sulfuric chlorohydrin is another strong acid widely used to produce primarily alcohol sulfates, alcohol ether sulfates, and dye intermediates. ClSO_3_H is an expensive source of SO_3_ (~US$ 0.255 per pound of reactive SO_3_), highly toxic, corrosive, rapid, and stoichiometric reactant that can be used as a dehydrating, oxidizing, and chlorinating agent. During its reaction, it liberates hydrochloric acid as a by-product, which is normally neutralized with water or dilute basic solution. In graphene research, ClSO_3_H has been used as a solvent for high concentration dispersion and expansion/exfoliation of graphitic domains under ambient conditions [106,107,108].

**Scheme 7:**
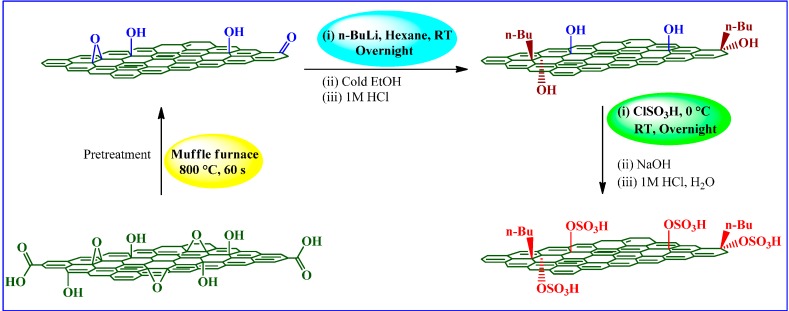
ClSO_3_H-induced sulfation of GO.

Taking into account the excellent nucleophilicity of organolithium compounds toward epoxy and carbonyl functions, Wang *et al.* [84] introduced a novel synthesis methodology for sulfated graphene under relatively mild conditions. As such, the sulfation steps of epoxide, hydroxyl and carbonyl functions were suggested to follow the mechanism shown in Scheme 7. These include pretreatment of GO, organolithiation of rGO with *n*-butyl lithium and sulfation of as-obtained chemically converted graphene (CCG).

The formation of O-S bonds in the as-prepared sulfated graphene was evidenced by FTIR spectroscopy, where a peak at 3691 cm^−1^ corresponding to the O-H stretching vibrations in CCG disappeared and reappeared (at 3450 cm^−1^) along with an additional peak at 1006 cm^−1^ (S=O stretching vibrations). The Raman spectra of rGO, CCG, and sulfated graphene indicated an *I*_D_/*I*_G_ ratio of 0.92, 0.84, and 0.94, respectively. The thermal stability of all samples was assayed by TG analysis and determined to be: rGO > sulfated graphene > CCG. The quantitative XPS analysis of sulfated graphene showed 2.36% S by mass, however, IEC was not taken into account by the authors [84].

In another work, Upare *et al.* [86] employed ClSO_3_H to functionalize commercial samples (Sigma-Aldrich, Germany) of GO and activated carbon with -SO_3_H groups. It has been claimed that as-prepared GO-SO_3_H catalyst can be used for the selective decomposition of hexose sugars into levulinic acid and other value-added derivatives. However, this report is somewhat difficult to harmonize in view of the reported experimental data. For instance, XRD pattern of GO are reported to be at 2θ = 26°; GO exhibits a sharp diffraction peak at 2θ = 10°–13°. We think that perhaps rGO is misinterpreted as GO in this report, as only the former gives diffraction patterns in the range from 2θ = 21.5° to 27.5°. Our assumption may be further linked to EA results demonstrating a very high C/O ratio of 14.12 for GO, which is quite unlikely. In fact, this value is very close to that observed (13.4) with NaBH_4_-induced reduction of graphite oxide [76].

Combined with a knowledge of sulfonation or sulfation chemistry, we can make an educated guess that the reactivity of ClSO_3_H towards -OH functions of GO should even be higher than that of with oleum. Nevertheless, chlorosulfonation reactions cannot be ruled out completely.

#### 3.2.4. Sulfuric Acid

Sulfuric acid (H_2_SO_4_), historically known as oil of vitriol, is a well-known strong mineral acid having significant oxidizing and dehydrating properties. In the chemical industry it has a central importance and is increasingly used for variety of applications such as in oil refining, fertilizer manufacturing, mineral and waste water processing, acidic drain cleaning, as well as in acid-catalyzed reactions as mentioned previously. In typical organic chemistry, it is used for sulfonation of benzene through an electrophilic aromatic substitution under reflux conditions. However, the sulfonation with H_2_SO_4_ can be considered as a special case of oleum sulfonaion. In present scenario, the H_2_SO_4_ sulfonation is principally used for production of hydrotropes by azeotropic reaction with toluene, xylene, or benzene.

Recently, Antunes *et al.* [89] have reported the use of H_2_SO_4_ to introduce sulfur-containing acid groups onto the graphene surface. Unlike previous studies, GO (in conjunction with CNTs and carbon black (CB)) was reduced by high temperature (190 °C) treatment with benzyl alcohol under microwave irradiation. The as-obtained rGO (coined as RGO in the original report) was subjected to H_2_SO_4_ (97 wt %) treatment at 160 °C for 5 h and this process was repeated twice more. The BET surface area of the as-obtained material (S-RGO) was determined to be 12 m^2^·g^−1^; far below than those of S-CNTs (44 m^2^·g^−1^) and S-CB (228 m^2^·g^−1^). As described above, the Raman results were consistent and an increase in *I*_D_/*I*_G_ ratio was observed in all CNMs after H_2_SO_4_ treatment.

**Figure 4:**
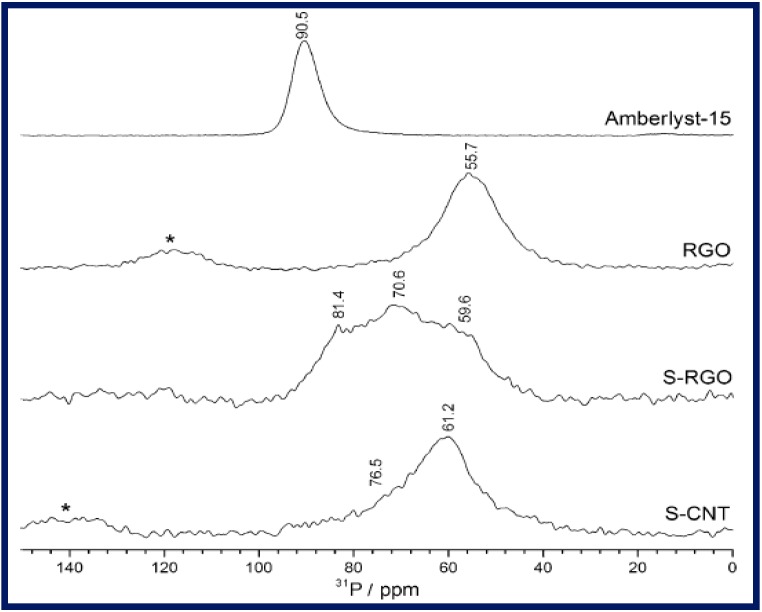
The ^31^P MAS NMR spectra of TEPO adsorbed onto carbocatalysts and Amberlyst-15. Reproduced with permission from [89]. Copyright (2014) Wiley-VCH Verlag GmbH & Co.

The acid strength of S-RGO in combination with rGO, Amberlyst-15, and S-CNTs was assayed by ^31^P magic-angle spinning (MAS) NMR spectroscopy using triethylphosphine oxide (TEPO) as a basic probe. As shown in Figure 4, the appearance of signals at 55–60 ppm in both rGO and S-RGO were attributed to the presence of non-sulfur acid groups while the latter gave additional signals at higher chemical shifts (71–80 ppm), possibly due to the presence of sulfur species. As expected, the sulfonic acid-based cation exchange resin, Amberlyst™-15 (referred to as Amberlyst-15 in ref. [89]), displayed a single sharp resonance ~90 ppm demonstrating its stronger acidity than that of carbocatalysts.

The total acid content of S-RGO as measured by acid-base titrations was determined to be 2.2 H^+^ mmol·g^−1^ and was attributed to the presence of -COOH and -OH groups. On the other hand, EA results indicated relatively low sulfur content (1.08 mmol·g^−1^) which was credited to the presence of sulfonic acids. However, the TGA outcome is in contrast to this hypothesis. In particular, a second weight loss in TGA is observed in between 230–320 °C; far below the decomposition temperature (~500 °C) of sulfonic acid groups [57]. As such, one can assume that rather than sulfonic these are hydrosulfate groups that may be generated during oxidative treatment of rGO with H_2_SO_4_. In this regard, a comparison between S-RGO and GO, especially, ^31^P MAS NMR experimental data is not only desirable but also would be praiseworthy.

Aside from the above-described sulfonating agents, He *et al.* [88] have reported the use of ammonium sulfate ((NH_4_)_2_SO_4_) in the simultaneous reduction and sulfonation of GO. It was demonstrated that when a mixture of GO and (NH_4_)_2_SO_4_ (in mass ratio of 5:1) was dried by lyophilization and subsequently heated at 235 °C in an inert atmosphere, the sulfonic acid groups could be grafted onto GO, whereas GO could be simultaneously reduced. The as-obtained material (S-rGO) was characterized by SEM, EDX, IR and Raman spectroscopy and used as support for metal nanocatalysts. The acid-density of S-rGO in terms of equivalent weight was evaluated to be 727 ± 5 g·mol^−1^.

It is worthy of mention here that despite the efforts of many chemists since the 18th century [109,110,111,112], the exact mechanism of thermal decomposition of (NH_4_)_2_SO_4_ remains elusive. Nevertheless, the use of (NH_4_)_2_SO_4_ as a sulfonating agent, as far as we are aware, is not common in practice, presumably, due to its most feasible two-steps decomposition pathway [113] as shown in Equations (1) and (2):

2(NH_4_)_2_SO_4_ → (NH_4_)_2_S_2_O_7_ + 2NH_3_ + H_2_O (1)

3(NH_4_)_2_S_2_O_7_ → 2NH_3_ + 6SO_2_ + 2N_2_ + 9H_2_O (2)

(NH_4_)_2_SO_4_ → 2NH_3_ + SO_3_ + H_2_O (3)

The ammonium pyrosulphate ((NH_4_)_2_S_2_O_7_), the primary condensation phase product (Equation (1)) is generally obtained at temperatures in between 100–250 °C while the reaction shown in Equation (2) occurs at relatively high temperature (>250 °C). Thus, the only possibility of generating SO_3_ is the likely reaction of SO_2_ with oxygen at relatively high temperature. Though a similar (NH_4_)_2_SO_4_-induced sulfonation protocol was also reported previously [114], we believe that characterization data is insufficient, leading to confusion in understanding the nature of this reaction. Furthermore, the acidity is commonly presumed and interpreted from the perspective of sulfur content in -SO_3_H groups. Now, if we assume that the decomposition pathway does exist in accordance with Equation (3), is this process gentle enough to leave other molecules, such as ammonia, untouched?

Indeed, the thermal decomposition of (NH_4_)_2_SO_4_ within graphene domain appears to us quite interesting and it is quite reasonable to expect that some interesting materials can be formed simply by switching the experimental conditions.

## 4. Applications of G-NMs in Heterogeneous Acid Catalysis

Acid-catalyzed reactions are classified according to the type of acid catalysts. The catalytic performances of G-NMs in conjunction with other solid/liquid catalysts have been discussed in terms of catalyst loadings, products yield, recoverability, and recyclability at certain instances.

### 4.1. Graphite Oxide as Acid Catalyst

#### 4.1.1. Michael-Type Friedel-Crafts Addition

**Scheme 8:**
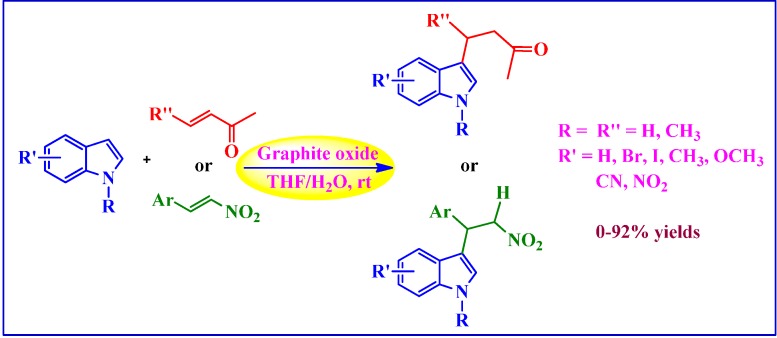
Graphite oxide catalyzed Michael-type Friedel-Crafts addition of indoles.

Intrigued by the strong acidity of graphite oxide, Kumar and Rao [115] used it as catalyst in the Friedel-Crafts addition of indoles to α,β-unsaturated ketones as well as nitrostyrenes (Scheme 8). The superiority of graphite oxide over other acid catalysts including HCl, H_2_SO_4_, acidic Al_2_O_3_, nano MgO/CuO/Fe_2_O_3_, (sulfo)β-cyclodextrins, activated charcoal, and *p*-TSA was demonstrated by addition of indole to methyl vinyl ketone in THF-H_2_O mixtures at ambient conditions. Whereas graphite oxide (20 wt %) afforded excellent yield (92%), the other catalysts only gave poorer to moderate yields (5%–67%) of addition product. For the same reaction, graphite oxide could be recycled up to five times without apparent loss in catalytic activity. Indoles with electron-donating and electron-withdrawing substituents underwent reactions smoothly in the addition to methyl vinyl ketone with no polymerized or dimerized side products. However, no reaction was observed with carboxylic acid/ester substituents, even after 24 h. Furthermore, the reaction of indole with electron deficient nitrostyrene took quite a longer time to afford products in moderate yields.

#### 4.1.2. Polymerization

Inspired by their initial discovery of GO’s acidity [30], Dreyer *et al.* [116] used graphite oxide (2.5–20 wt % loadings) to catalyze the ring opening polymerization of various cyclic lactones and lactams to their corresponding carbon-reinforced, high molecular weight polyesters, or polyamide composites in good to excellent yields (39%–100%). In contrast to most other methods, which often rely on clumsy two-step protocols‒preparing the polymer followed by mixing of the carbon filler, the use of a graphite oxide combines the polymerization and dispersion of the filler into a single expeditious step.

#### 4.1.3. Synthesis of Dipyrromethanes

Chauhan *et al.* [117] examined the reactions of pyrrole and ketones using graphite oxide as acid catalyst under a variety of conditions. Specifically, moderate to high yields of 5,5-dialkyldipyrromethanes were achieved along with linear oligomers and cyclic tetramers under ambient conditions.

### 4.2. Graphene Oxide (GO) as Acid Catalyst

#### 4.2.1. Hydration of Alkynes

**Scheme 9:**
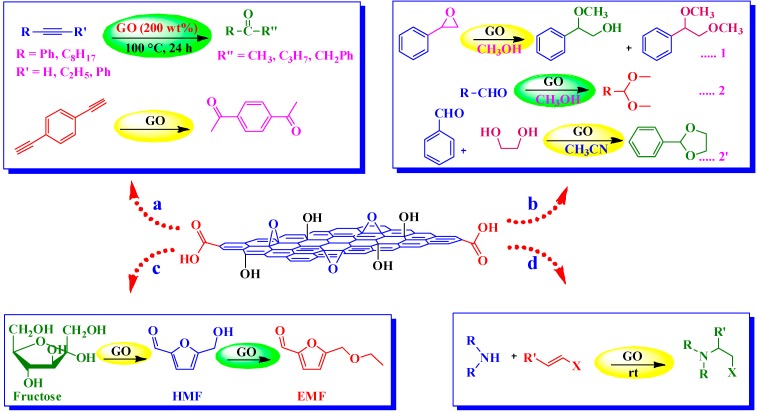
GO catalyzed (**a**) Hydration of alkynes. (**b**) Ring opening of epoxides and acetalization of aldehydes. (**c**) Conversion of carbohydrates into 5-ethoxymethylfurfural (EMF). (**d**) Aza-Michael addition.

The first use of GO [30] as an acid catalyst was demonstrated in the hydration of various alkynes to their corresponding ketones (Scheme 9a). In particular, in the presence of GO, low to excellent conversions of alkynes (27%–98%) were achieved at 100 °C as monitored by ^1^H-NMR spectroscopy. However, the need to use significantly high GO loadings (200 wt %) in these reactions somewhat limits the practical utility of this protocol.

#### 4.2.2. Aza-Michael Addition

The Aza-Michael addition of amines to activated alkenes in the presence of GO catalyst furnishing β-amino compounds was reported by Verma *et al.* [118] (Scheme 9d). The reactions were found to proceed under relatively mild conditions and to afford excellent product yields (70%–97%) within short reaction times. The GO was recyclable up to nine runs with the consistent catalytic activity.

#### 4.2.3. Ring Opening of Epoxides and Acetalization of Aldehydes

Dhakshinamoorthy *et al.* [53] employed GO as an acid catalyst in the ring opening of epoxides with methanol and other primary alcohols as nucleophile and solvents. Under ambient conditions, the product selectivity could be switched by changing the reaction time. However, most of the reactions exhibited high regioselectivity rendering the product expected from S_N_1 mechanism. Interestingly, the conversion and selectivity in the presence of GO were found comparable to those of H_2_SO_4_ and *p*-TSA and much higher than Norit A, activated carbon, as demonstrated by styrene oxide with methanol as a model reaction (Scheme 9b-1). The high activity of GO was attributed to the presence of hydrogen sulfate groups on GO surface. The catalyst could be easily recovered after the reaction and successfully reused for up to three cycles. In their subsequent work [119], the group further examined the activity of GO for the acetalization of aldehydes in methanol (Scheme 9b-2). The GO-induced reaction of a variety of aromatic and aliphatic aldehydes with methanol afforded the corresponding acetalized products in moderate to excellent yields as determined by GC. In the case of 4-methylbenzaldehyde, the low yield (10%) was attributed to the poisoning of GO due to the formation of toluic acid, while 4-pyridylcarboxaldehyde and acetophenone were completely unreactive. The protocol was further extended successfully to the acetalization of benzaldehyde with ethylene glycol (Scheme 9b-2').

#### 4.2.4. Conversion of Carbohydrates into 5-Ethoxymethylfurfural (EMF)

Wang *et al.* [54] discovered that GO can be used as a facile acid catalyst for the one-step conversion of fructose-based carbohydrates into high-heating value liquid biofuels (Scheme 9c). In this regard, one-pot conversions of sucrose, inulin, and fructose into EMF using DMSO-ethanol mixtures (1:2.3, v/v) were achieved with yields of 34%, 66%, and 71%, respectively. Interestingly, GO-catalyzed direct etherification of 5-hydroxymethyl furfural (HMF), a dehydration product of carbohydrates, in ethanol afforded high EMF yield of 92% as determined by LC/MS. On the contrary, traditional liquid and solid acid catalysts such as Amberlyst-15, *p*-TSA, and H_2_SO_4_ gave only moderate yields of EMF (54%–61%) even though with 100% conversions as determined by NMR. Though a high yield of EMF (82%) was detected with H_3_PW_12_O_40_, it was dissolved in the reaction mixture and couldn’t be separated. The high activity of GO was attributed to the presence of hydrogen sulfate groups as mentioned above and found consistent even after several runs.

### 4.3. Sulfonated/Sulfated Graphene and Graphene Oxide (GO) as Acid Catalysts

#### 4.3.1. Sulfonated Graphene and GO as Acid Catalysts Prepared by Diazonium Salt

##### Hydrolysis of Ethyl Acetate

Ji *et al.* [82] used sulfonated graphene in the water participating reaction, hydrolysis of ethyl acetate. To evaluate its activity and stability, H_2_SO_4_ and Nafion NR50 catalysts were employed as controls. Whereas Nafion NR50 showed poorer activity, the activity of sulfonated graphene was comparable to that of H_2_SO_4_ and remain unchanged even after five consecutive runs.

##### Synthesis of Xanthenes and Benzoxanthenes

**Scheme 10:**
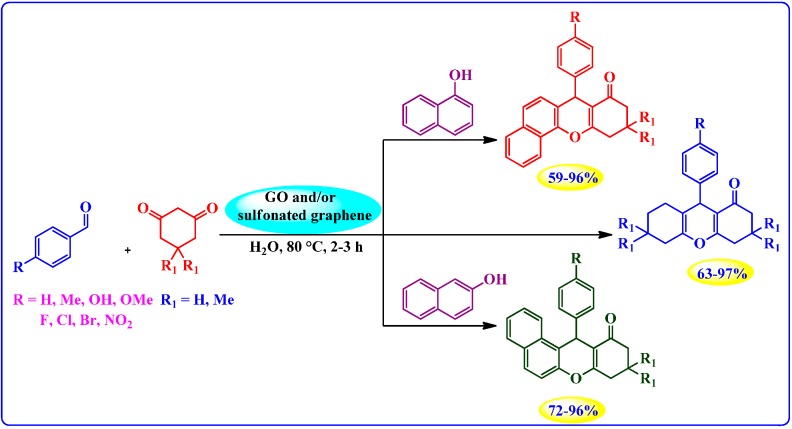
GO and/or sulfonated graphene catalyzed synthesis of xanthenes and benzoxanthenes in water.

Shaabani *et al.* [85] used sulfonated graphene in conjunction with GO for the one-pot condensation of aldehydes, 1,3-diketones, and naphthols into the corresponding xanthenes and benzoxanthenes (Scheme 10). 

Unlike InCl_3_, P_2_O_5_, HY zeolite, and Sr(OTF)_2_, the G-NMs exhibited high catalytic activity in water and could be reused several times; the best results were obtained with sulfonated graphene.

##### Dehydration of Xylose

**Scheme 11:**
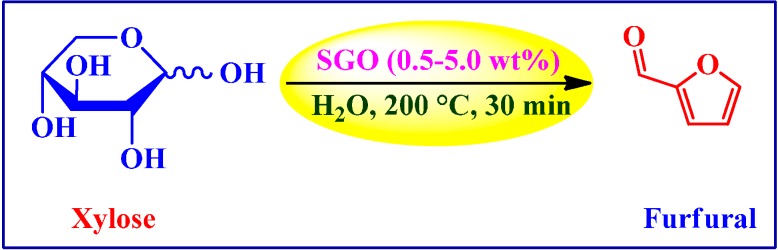
Sulfonated GO catalyzed dehydration of xylose into furfural.

Lam *et al.* [87] applied sulfonated GO (SGO) and graphene in conjunction with unfunctionalized GO and graphene for the dehydration of xylose to furfural (Scheme 11). Out of the four tested catalysts (2 wt % loadings, 35 min), SGO afforded highest yield of furfural (62%) with 75% selectivity as averaged over three runs. On the other hand, yields determined in the absence of catalyst as well as in the presence of graphene, GO, and sulfonated graphene were in the following order: sulfonated graphene (55%) > GO (53%) > graphene (51%) > no catalyst (44%). Interestingly, at only 0.5 wt % loading *vs.* xylose, SGO could be reused over multiple runs maintaining an average yield of 61%, despite the presence of accumulated byproducts on its surface. It has been suggested that strongly acidic aryl sulfonic acid groups in SGO are the key active sites for the high temperature production of furfural in water.

#### 4.3.2. Sulfonated Graphene as Acid Catalyst Prepared by Fuming Sulfuric Acid or Oleum

##### Esterification, Peckmann, and Hydration Reactions

**Scheme 12:**
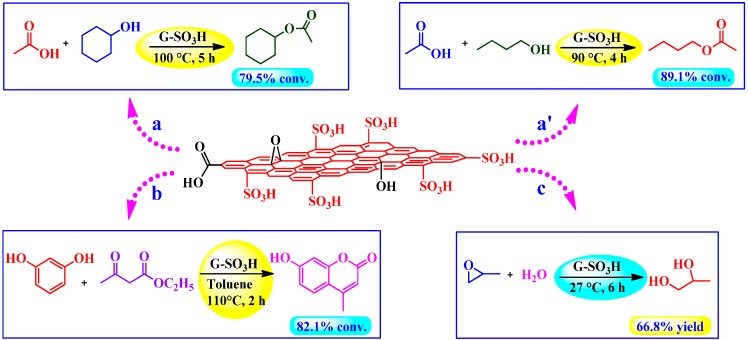
G-SO_3_H catalyzed (**a**) Esterification of acetic acid with cyclohexanol. (**a’**) Esterification of acetic acid with *n*-butanol. (**b**) Peckmann reaction of resorcinol with ethyl acetoacetate. (**c**) Hydration of propylene oxide.

Liu *et al.* [83] employed sulfonated graphene (G-SO_3_H) as solid catalyst in a number of acid-catalyzed reactions including esterification, hydration, and Peckmann reactions (Scheme 12). The esterification reactions of acetic acid with cyclohexanol and *n*-butanol in the presence of G-SO_3_H (after pretreatment with 0.1 M H_2_SO_4_) afforded the corresponding esters with the highest conversions of ~79% and 89%, respectively (Scheme 12a,a’). Furthermore, G-SO_3_H exhibited almost similar activity when reactions were performed under static conditions. On the contrary, the conversions were significantly low (<53% and <76% for cyclohexanol and *n*-butanol, respectively) in the presence of other solid catalysts such as OMC-SO_3_H, rGO and SBA-15-SO_3_H. Nevertheless, Amberlyst-15 gave second highest conversions for the same. Notably, in contrast to available reports, GO showed poorer catalytic activity with as low as <22% conversions. Surprisingly, no acid sites were detected in GO as measured by acid-base titrations and S analysis.

In yet another example, G-SO_3_H catalyzed Peckmann reaction of resorcinol with ethyl acetoacetate in toluene afforded corresponding coumarin with ~82% conversion (Scheme 12b). On the other hand, conversions were decreased significantly in the presence of other catalysts and determined in the following order: Amberlyst-15 (~75%) > OMC-SO_3_H > SBA-15-SO_3_H >> GO > rGO (~11%). A similar trend was recognized in the hydration of propylene oxide (Scheme 12c), however, GO and rGO were found completely inactive.

The recyclability of G-SO_3_H was examined in association with OMC-SO_3_H. After being recycled five times, G-SO_3_H gave relatively higher conversions than OMC-SO_3_H. The activity loss in G-SO_3_H was 1.5%–2.8% whereas OMC-SO_3_H had a significant reduction in activities (~7%–10%) after five cycles. The high stability and activity of G-SO_3_H were attributed to the presence of stable sulfonic acid groups on its surface and unlimited mass transfer owing to its unique sheet structure.

#### 4.3.3. Sulfated Graphene as Acid Catalyst Prepared by ClSO_3_H

##### Ester-Exchange Reactions

Wang *et al.* [84] evaluated the catalytic efficiency of sulfated graphene for the ester-exchange reaction between ethyl acetate and a variety of alcohols. Specifically, sulfated graphene catalyzed reaction of alcohols gave corresponding esters in ≥96% isolated yield; somewhat superior to that of Dowex 50 W × 2 (50–100 mesh; 95% yield). It was postulated that nucleophilicity of alcohols play a significant role in ester-exchange reactions. The catalyst could be recycled up to three times.

##### Chemical Conversion of Carbohydrates to Levulinic Acid (LA)

**Scheme 13:**
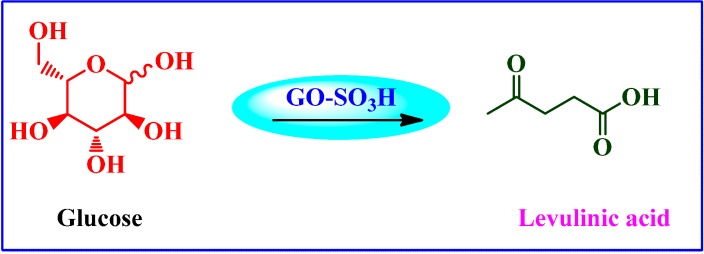
Chemical conversion of glucose to levulinic acid (LA).

Upare *et al.* [86] used -SO_3_H functional GO catalyst in conjunction with activated carbon for the selective decomposition of glucose and fructose into levulinic acid (LA) (Scheme 13). The graphene-based catalyst gave high yield of LA (78%) and showed good reusability with reliable performance. It was postulated that chemical transformation patterns for hexose sugar decompositions are affected by the type of catalyst, the density of acid sites as well as temperature. In addition, the presence of other functional groups including -COOH and -OH groups on catalyst surface help in enhancing the adsorption of sugar for the reaction.

#### 4.3.4. Sulfated rGO as Acid Catalyst Prepared by H_2_SO_4_

##### Conversion of HMF into Biofuels

**Scheme 14:**
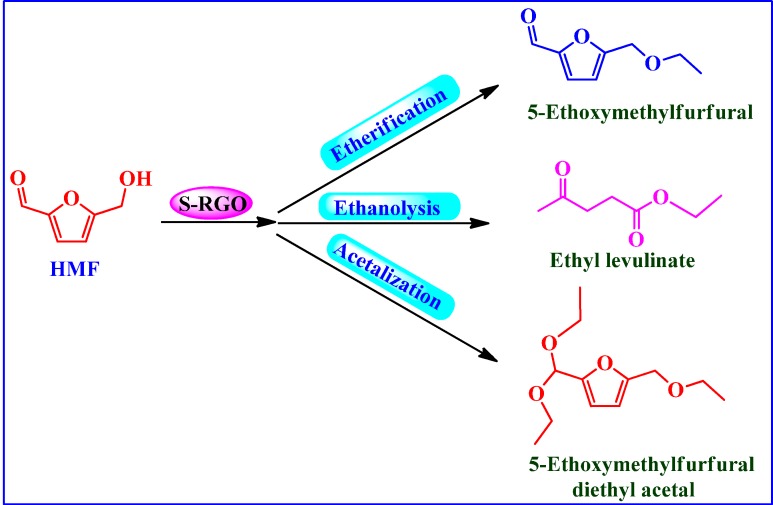
Conversion of 5-hydroxymethylfurfural (HMF) into value-added biofuels.

Antunes *et al.* [89] used H_2_SO_4_-treated rGO (S-RGO) as potential catalyst in the conversion of 5-hydroxymethylfurfural (HMF) to biofuels (Scheme 14) such as 5-ethoxymethylfurfural (EMF), 5-(ethoxymethyl)furfural diethyl acetal (EMF da), and ethyl levulinate (EL). The catalytic performance of S-RGO was found superior to that of H_2_SO_4_ treated carbon black, CNTs as well as Amberlyst-15 and was attributed to the different acid sites and 2D structure of the catalyst. The catalyst could be reused up to three cycles without significant loss in catalytic activity.

## 5. Conclusions: Final Remarks

As the building block of CNMs of all dimensions, graphene possesses many unique properties and physicochemistry. In this comprehensive review, we have shed light on graphite oxide, GO, and their sulfonated or sulfated derivatives acting as main performers in heterogeneous acid catalysis. The observation that graphite oxide and GO, prepared by a modified Hummer’s method, convey inherent acidic character has motivated scientists to get new insights into the structural properties of graphite oxidation products. In this regard, new theories and approaches are constantly being explored and somewhat mystifying the commonly accepted LK model. Another important property of GO is its easy functionalization rendering it useful in preparing a library of catalytically active sulfonated or sulfated NMs. Indeed, the heterogeneous acid catalysis using these NMs has the potential to undergo a renaissance and to address many industrial processes not previously accessible to it.

Despite this, a basic understanding of working actions of sulfonating agents on (r)GO is still in its infancy, which have resulted in conceptual vagueness in many aspects. Furthermore, due to the lack of comparative yet solid experimental data of starting (GO) and final products (sulfonated or sulfated G-NMs), a more precise acid activity has not been ascertained. These shortcoming are of utmost importance since two key concerns may plague future scientists interested in using sulfonation methods, mainly whether a sulfonating agent of interest would: (i) specifically target aromatic carbon (sulfonation) or -OH functions (sulfation) in a certain mode of reaction and (ii) undergo a random mode of reaction. In this context, a fundamental understanding of sulfonation chemistry combined with routine industrial experience can only act as a guide for advancements in this area.

As a final note, while increasingly tight legislation on the control of chemical effluents currently at play, heterogeneous acid catalysis with G-NMs holds great promise to achieve the objectives of industrial catalysis following the principles of sustainable and green chemistry. We postulate that by the mutual cooperation between chemists, physicists and material scientists, graphene-based heterogeneous acid catalysis may lead to new disciplines in the 21st century. It is just a matter of patience and constant efforts to discover new catalysts or novel methodologies; after all, they are also patiently waiting to be discovered.
